# Challenges associated with the targeted delivery of gelonin to claudin-expressing cancer cells with the use of activatable cell penetrating peptides to enhance potency

**DOI:** 10.1186/1471-2407-11-61

**Published:** 2011-02-08

**Authors:** Xiaoqin Yuan, Xinjian Lin, Gerald Manorek, Stephen B Howell

**Affiliations:** 1 Department of Medicine and the Rebecca and John Moores UCSD Cancer Center, University of California-San Diego, 3855 Health Sciences Drive, La Jolla, CA 92093-0819, USA

## Abstract

**Background:**

Treatment of tumors with macromolecular toxins directed to cytoplasmic targets requires selective endocytosis followed by release of intact toxin from the endosomal/lysosomal compartment. The latter step remains a particular challenge. Claudins 3 and 4 are tight junction proteins that are over-expressed in many types of tumors. This study utilized the C-terminal 30 amino acid fragment of *C. perfringens *enterotoxin (CPE), which binds to claudins 3 and 4, to deliver a toxin in the form of recombinant gelonin (rGel) to the cytoplasm of the human ovarian carcinoma cell line 2008.

**Results:**

CPE was fused to rGel at its N-terminal end via a flexible G_4_S linker. This CPE-G_4_S-rGel molecule was internalized into vesicles from which location it produced little cytotoxicity. To enhance release from the endosomal/lysosomal compartment a poly-arginine sequence (R_9_) was introduced between the CPE and the rGel. CPE-R_9_-rGel was 10-fold more cytotoxic but selectivity for claudin-expressing cells was lost. The addition of a poly-glutamic acid sequence (E_9_) through a G_4_S linker to R_9_-rGel (E_9_-G_4_S-R_9_-rGel) largely neutralized the non-selective cell membrane penetrating activity of the R_9 _motif. However, introduction of CPE to the E_9_-G_4_S-R_9_-rGel fusion protein (CPE-E_9_-G_4_S-R_9_-rGel) further reduced its cytotoxic effect. Treatment with the endosomolytic reagent chloroquine increased the cytotoxicity of CPE-E_9_-G_4_S-R_9_-rGel. Several types of linkers susceptible to cleavage by furin and endosomal cathepsin B were tested for their ability to enhance R_9_-rGel release but none of these modifications further enhanced the cytotoxicity of CPE-E_9_-G_4_S-R_9_-rGel.

**Conclusion:**

We conclude that while a claudin-3 and -4 ligand serves to deliver rGel into 2008 cells the delivered molecules were entrapped in intracellular vesicles. Incorporation of R_9 _non-specifically increased rGel cytotoxicity and this effect could be masked by inclusion of an E9 sequence. However, the putative protease cleavable sequences tested were inadequate for release of R_9_-rGel from CPE-E_9_-G_4_S-R_9_-rGel.

## Background

The claudin (CLDN) family of transmembrane proteins plays an integral role in the formation and function of tight junctions. Using gene expression profiling, we and others have found that claudin-3 (*CLDN3*) and claudin-4 (*CLDN4*) genes are highly expressed in ovarian cancers [1-3]. In addition, several other studies have reported aberrant claudin expression in various cancers. Some examples include increased expression of *CLDN3 *and *CLDN4 *in prostate and uterine cancers [4,5], and high *CLDN4 *expression in pancreatic cancer [6,7]. These two genes are not normally highly expressed in non-malignant human tissues including the normal ovarian epithelium [8], clearly associating abundance of these two proteins with malignancy. Although their functional role in cancer development and progression remains unclear, the differential expression of these proteins between tumor and normal cells makes them prime candidates for cancer targeted therapy [9]. Preclinical studies have shown that tumor cells over-expressing CLDNs can be successfully targeted both *in vitro *and *in vivo *by a fusion protein composed of the C-terminal fragment (amino acids 184 to 319) of *Clostridium perfringens *enterotoxin (CPE), a natural ligand for CLDNs, and the protein synthesis inhibitory factor (PSIF) which lacks the cell binding domain of *Pseudomonas *exotoxin [10,11]. When CPE binds to CLDNs it triggers endocytosis most likely via a clathrin-dependent process. We previously reported *in vitro *characterization of a fusion protein, CPE_290-319_-TNF, and demonstrated that the C-terminal 30 amino acids (amino acids 290-319) of CPE could effectively target TNF to ovarian cancer cells expressing claudin-3 and claudin-4 [12].

Gelonin (rGel) is a class I ribosome-inactivating protein derived from the plant *Gelonium multiforum*. Similar in action to other plant toxins such as ricin, gelonin induces cell death by removing the base A4324 in 28 s rRNA which prevents the association of elongation factor-1 and -2 (EF-1 and EF-2) with the 60 s ribosomal subunit, eventually causing cell death in eukaryotic cells [13]. Since gelonin functions enzymatically, only a few molecules are needed to kill a cell, but by itself gelonin has very limited toxicity because it is not able to cross the plasma membrane at levels that are therapeutically useful. This has prompted the development of strategies to improve intracellular accumulation. Gelonin has been used to construct a large number of different kinds of immunotoxins, some of which are currently undergoing clinical testing [14-16].

Cancer therapies that exploit targeting ligands to deliver attached cytotoxic drugs selectively to malignant cells are currently receiving significant attention. However, the lipophilic nature of the biological membranes restricts the direct intracellular delivery of such compounds. While some short peptides can enter cells, the cell membrane prevents large molecules, such as proteins and DNA, from entering cells unless there is an active transport mechanism. Under certain circumstances these molecules, or even small particles, can be transferred from the extracellular space into cells by the receptor-mediated endocytosis. However, the problem is that most molecules or particles entering the cell via the endocytic pathway become entrapped in endosomes and eventually get degraded in the lysosomal compartment. As a result, only a small fraction of active material reaches the cytoplasm. It has been reported that a poly-arginine tract such as R_9_, which is also a furin cleavage site, can aid in translocating a recombinant pro-apoptotic protein targeting the HER2 receptor from the endosomal to the cytosolic compartment leading to enhanced cell killing activity [17]. However, due to the fact that all the arginine-rich cell-penetrating peptides (CPPs) induce a strong non-specific cell binding, they lack cell specificity and this remains the major impediment to development. Tsien and coworkers [18] previously developed a new strategy, designated "activatable cell penetrating peptides (ACPP)" by which the cellular association of the positively charged R_9 _is effectively blocked by fusing it to a domain made up of negatively charged glutamates (E_9_) via a cleavable linker. Adsorption and cellular uptake of the CPP portion and its attached cargo are inhibited until the linker is cleaved by a tumor protease. In the present study, we describe the construction and characterization of several rGel-based chimeric toxins composed of different combinations of CPE_290-319_, R_9 _and E_9_. We have used these to examine how these modules affect the internalization and cytotoxic activity when tested against CLDN-expressing ovarian cancerous cells.

## Methods

### Reagents

Tissue culture media were purchased from Life Technologies (Frederick, MD), pE-SUMOstar vector and SUMOstar protease 1 from LifeSensors, Inc (Malvern, PA), and metal-affinity resin Ni-NTA agarose from Qiagen (Valencia, CA). Rabbit anti-gelonin antibody was a gift from Dr. Michael G. Rosenblum (MD Anderson Cancer Center, Houston, TX). Texas red-labeled secondary antibodies against rabbit immunoglobulin were obtained from Jackson ImmunoResearch Laboratories, Inc (West Grove, PA).

### Cells and cell culture

The human ovarian carcinoma cell line 2008 that expresses both CLDN3 and CLDN4 and its CLDN3 knockdown subline 2008-CLDN3KD-4.5 [12] were grown in RPMI 1640 supplemented with 5% fetal bovine serum. Cultures were maintained at 37°C in a humidified atmosphere of 5% CO_2 _and 95% air.

### Plasmid construction

The gene encoding gelonin in the pET-32 bacterial expression vector was kindly supplied by Michael G. Rosenblum [19]. Recombinant fusion toxins containing rGel and either CPE_390-319 _or E_9_/R_9 _with the flexible linker (GGGGS, designated "G_4_S") were constructed by splicing overlap extension PCR. All the fusion genes were cloned into the T7 promoter-based *E. coli *expression vector pE-SUMOstar. All the constructed plasmids were sequence-verified and transformed into *E. coli *strain BL21(DE3) pLysS.

### Protein expression in *E. coli*

To express the recombinant fusion proteins, bacterial cultures were incubated at 37°C in LB growth medium with 200 μg/mL ampicillin and grown to log phase (A_600 _= 0.8). The target protein expression was induced at 18°C with 0.1 mmol/L isopropyl-Lthio-β-D-galactopyranoside for 6 h. Induced bacterial cultures were then centrifuged and stored frozen at -20°C overnight.

### Isolation and purification of fusion toxins

Frozen bacterial pellets were allowed to thaw with the addition of 50 mmol/L Na-phosphate (pH 7.6), 300 mmol/L NaCl, 10 mmol/L imidazole and 100 μg/ml lysozyme. The bacterial suspension was sonicated and then clarified by centrifugation at 14,000 g for 30 min at 4°C to pellet the cellular debris. The supernatant that contained the soluble fraction of the recombinant protein was loaded onto a Ni-NTA column pre-equilibrated with the same lysis buffer. After washing the column twice with a wash buffer (50 mM NaH_2_PO_4_, 300 mM NaCl and 20 mM imidazole, pH 8.0), the bound proteins were eluted with a elution buffer containing 50 mM NaH_2_PO_4_, 300 mM NaCl and 250 mM imidazole (pH 8.0). Absorbance (280 nm) and sodium dodecylsulfate polyacrylamide gel electrophoresis (SDS-PAGE) analyses were performed to determine which fraction(s) contained the majority of polyhistidine-tagged (6 × His tag) protein. Fractions were combined and dialyzed against 20 mM Tris-HCl (pH 8.0) and 150 mM NaCl, followed by digestion with SUMOstar protease 1 in the presence of 2 mM DTT. The final protein product was collected in the flow-through and buffer-exchanged with PBS using a PD-10 column.

### Reticulocyte lysate *in vitro *translation assay

The ability of rGel-containing toxins to inhibit [^3^H]leucine incorporation into a protein in a cell-free protein synthesizing system was assessed as described previously [20].

### Binding affinity

The binding affinity and specificity of rGel-based fusion toxins containing the combinations of CPE_290-319_, E_9 _and R_9 _were evaluated by ELISA on both CLDN3 and CLDN4-positive 2008 cells and CLDN3-knockdown cells. Rabbit anti-rGel antibody and horseradish peroxidase-conjugated goat anti-rabbit IgG was used as a tracer in this assay as described previously [21].

### Furin cleavage assay

Various rGel-based fusions containing different linkers were treated with recombinant human furin (New England Biolabs, Ipswich, MA) at pH 7.2. A dose of 2 units of purified furin was applied to 25 μg of fusion protein in each reaction. After incubation for 16 h at room temperature, the proteins were analyzed by SDS-PAGE with Coomassie blue staining.

### Internalization and intracellular distribution analyses

Immunofluorescence-based internalization studies were done on 2008 cells. Cells treated with relative fusion toxins were subjected to immunofluorescent staining with anti-rGel antibody followed by Texas red-conjugated secondary antibody. Microscopy was performed at the University of California San Diego Cancer Center Digital Imaging Shared Resource using a Zeiss LSM510 confocal microscope system (Carl Zeiss, Inc. Thornwood, NY). Images were captured from 0.8- μm sections by a 63× lens and analyzed by SoftWorx software (Applied Precision, Inc).

### *In vitro *cytotoxicity assay

The 2008 cells were plated into 96-well plates at a density of 2,000 cells/well and allowed to adhere overnight. The cells were then exposed to different concentrations of various toxins. After 96 h, the effects of the drugs on the growth of tumor cells in culture was determined by the same Cell Counting Kit-8 (CCK-8) method as described previously [12].

## Results

### Construction, expression and purification of rGel-based fusion proteins

**Figure 1:**
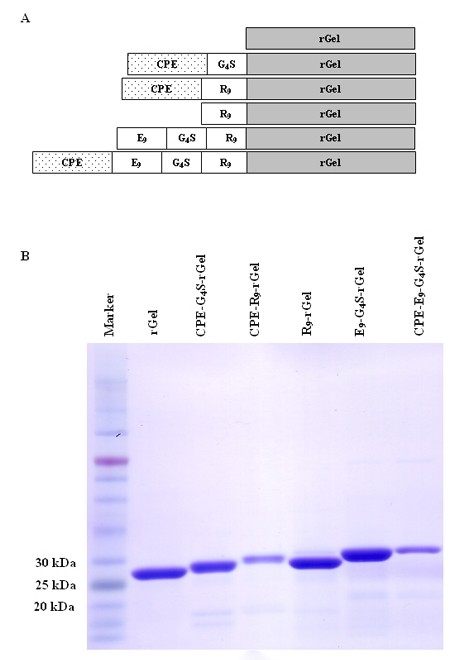
**Preparation of rGel-based series fusion toxins**. **A**. Schematic diagram of the fusion constructs containing CPE, E_9_, R_9 _and rGel. **B**. SDS-PAGE analysis of the final purified products of the above toxins.

As illustrated in Figure 1A, the initial rGel-based fusion toxins consisted of a flexible G_4_S linker tethering the C-terminus of CPE_290-319 _to the N-terminus of rGel. The 30 amino acid CPE_290-319 _peptide is abbreviated in this paper as "CPE". The CPE-G_4_S-rGel construct was further engineered by replacing G_4_S with the furin-cleavable cell-penetrating sequence R_9 _to make CPE-R_9_-rGel. Vectors expressing other variants containing combinations of CPE, E_9, _G_4_S and R_9 _were constructed by splicing overlap extension PCR (SOE-PCR). Each contained an N-terminal histidine tag to assist purification followed by a SUMO protease cleavage site that allowed subsequent removal of the histidine tag. Following expression in *E. coli*, purification and SUMO cleavage to remove the 6×His tag, the final purified products of all the rGel-based fusion toxins migrated on SDS-PAGE at the expected molecular weights of 28.2, 31.9, 33.0, 29.6, 31.3, and 34.4 kDa, respectively, for rGel, CPE-G_4_S-rGel, CPE-R_9_-rGel, R_9_-rGel, E_9_-G_4_S-R_9_-rGel and CPE-E_9_-G_4_S-R_9_-rGel (Figure 1B).

### Binding activity

**Figure 2:**
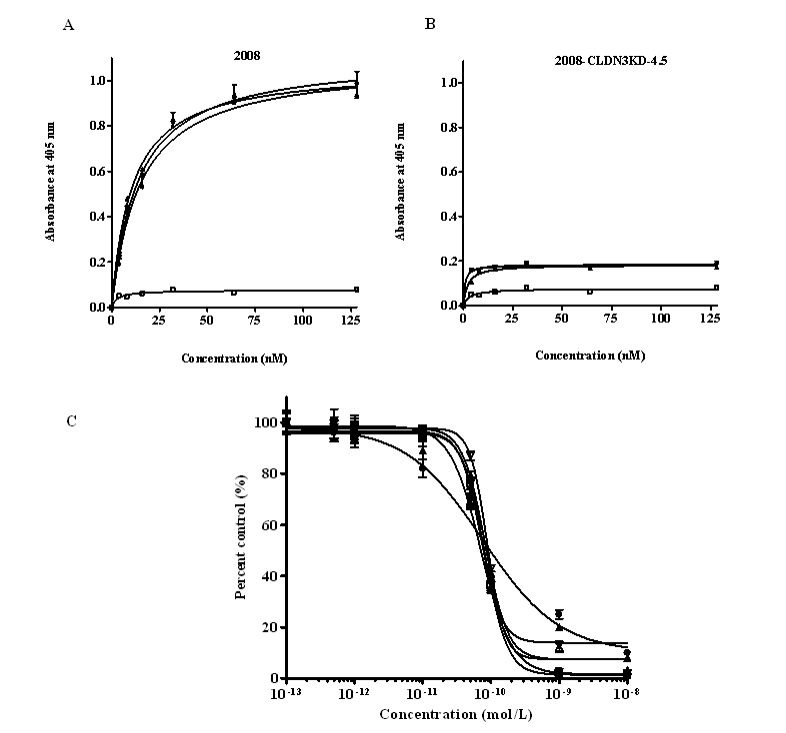
**Characterization and comparison of rGel-based fusion toxins**. **A**. Evaluation of the binding of the fusion constructs CPE-G_4_S-rGel (solid up-side-down triangle), CPE-R_9_-rGel (solid circle), CPE-E_9_-G_4_S-R_9_-rGel (solid upright triangle) and rGel (open square) to 2008 cells by whole-cell ELISA. **B**. Evaluation of the binding of the fusion constructs CPE-G_4_S-rGel (solid up-side-down triangle), CPE-R_9_-rGel (solid circle), CPE-E_9_-G_4_S-R_9_-rGel (solid upright triangle) and rGel (open square) to 2008-CLDN3KD-4.5 cells by whole-cell ELISA. **C**. The enzymatic (N-glycosidase) activity of the rGel component of the fusions (solid sqaure, rGel; solid upright triangle, CPE-G_4_S-rGel; solid circle, CPE-R_9_-rGel; open square, R_9_-rGel; open upright triangle, E_9_-G_4_S-R_9_-rGel; open up-side-down triangle, CPE-E_9_-G_4_S-R_9_-rGel) as assessed using rabbit reticulocyte lysate assay.

To ensure that the CPE-containing fusion toxins retained receptor binding, their ability to bind to a human ovarian carcinoma cell line was assessed using an ELISA-based binding assay (Figure 2A). The 2008 ovarian cancer cell line expresses both CLDN3 and CLDN4, and we have previously reported on the subline 2008-CLDN3KD-4.5, in which the expression of CLDN3 was knocked down using a lentiviral vector expressing a short hairpin RNA targeted to CLDN3 mRNA [12]. These isogenic CLDN3-positive and negative cells were exposed to graded concentrations of the fusion toxins, after washing the binding was assessed by determining the amount of cell-bound toxin by reaction with an anti-rGel antibody. Equilibrium dissociation constants were calculated using GraphPad Prism, v4.03. The affinity of CPE-G_4_S-rGel, CPE-R_9_-rGel, and CPE-E_9_-G_4_S-R_9_-rGel for CLDN-positive 2008 cells was similar with a K_d _value of 13.63, 14.73 and 10.57 nmol/L, respectively. The K_d _values are consistent with a previous report (Ka = 1.49 × 10^8 ^M^-1^) based on an ^125^I-CPE saturation binding assay [22]. In contrast, the binding of CPE-containing toxins to CLDN3-knockdown cells was markedly reduced (Figure 2B) suggesting that the binding specificity is dependent on the expression of the claudin receptors on the tumor cell membrane.

### Protein synthesis inhibitory activity

The biological activity of toxins can be severely compromised when incorporated into fusion constructs. To examine the N-glycosidic activity of the rGel component of the fusion toxins, these materials were added to an *in vitro *protein translation assay in which [^3^H] leucine is incorporated into isolated rabbit reticulocytes. Inhibition curves for the fusion constructs CPE-G_4_S-rGel, CPE-R_9_-rGel, R_9_-rGel, E_9_-G_4_S-R_9_-rGel and CPE-E_9_-G_4_S-R_9_-rGel were compared to that of native rGel. As shown in Figure 2C, the IC_50 _values for the six molecules were found to be virtually identical (71.2, 71.6, 75.6, 81.0, 82.2, 88.4 pmol/L, respectively, for rGel, CPE-G_4_S-rGel, CPE-R_9_-rGel, R_9_-rGel, E_9_-G_4_S-R_9_-rGel and CPE-E_9_-G_4_S-R_9_-rGel) although the shape of the inhibition curve for CPE-R_9_-rGel was slightly different from the others indicating that no loss of toxin activity occurred as a result of fusing rGel to the targeting moieties.

### *In vitro *cleavage of rGel-based toxins by furin

**Figure 3:**
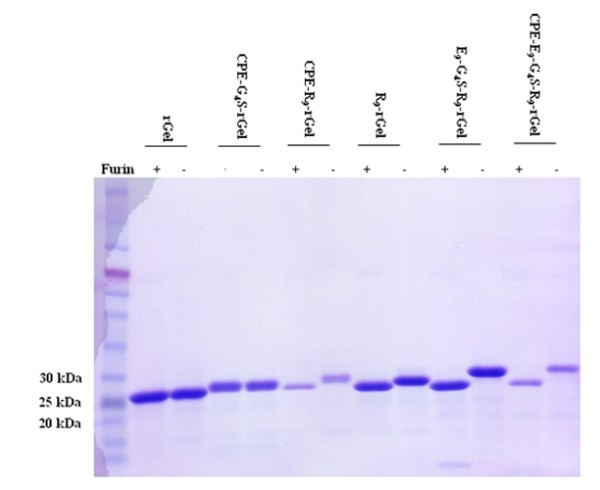
**SDS-PAGE analysis of *in vitro *furin cleavage of rGel-based fusion toxins**. Purified proteins were incubated with recombinant furin at room temperature for 16 h at pH 7.2.

To investigate the susceptibility of various chimeric toxins to proteolytic cleavage, the purified fusion proteins were subjected to proteolysis with recombinant furin in a physiological buffer at pH 7.2. As shown in Figure 3, CPE-R_9_-rGel, R_9_-rGel, E_9_-G_4_S-R_9_-rGel and CPE-E_9_-G_4_S-R_9_-rGel were all cleaved efficiently. In contrast, CPE-G_4_S-rGel, in which CPE is fused with rGel through a G_4_S linker was not cleaved. Thus, the R_9 _linker was sensitive to furin cleavage even at pH 7.2 whereas the G_4_S was found to be resistant to the action of this protease.

### Stability analysis and cellular uptake of rGel-based toxins

**Figure 4:**
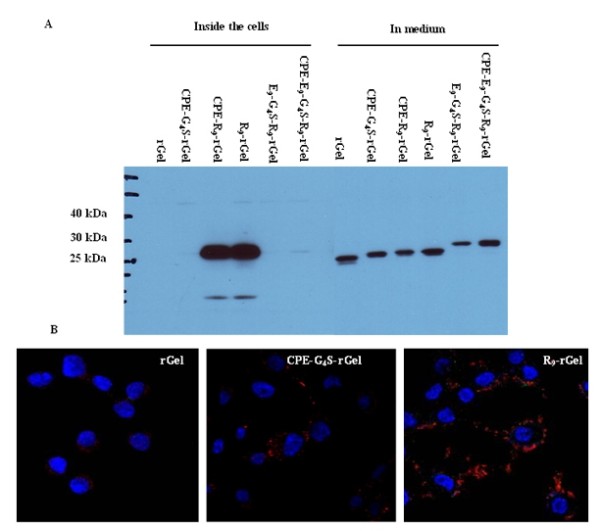
**Stability analysis and intracellular uptake of rGel fusion toxins**. **A**. Extent of degradation following exposure of 1 μM to 2008 cells for 6 h. The medium and cells were subjected to Western blot analysis with anti-rGel antibody. **B**. Internalization of rGel, CPE-G_4_S-rGel and R_9_-rGel into 2008 cells. Cells were treated with 250 nM of each fusion toxin for 6 h and stained with rabbit anti-gelonin antibody and Texas red-coupled anti-rabbit secondary antibody. Nuclei were stained with Hoechst 33342 (blue).

Stability of various rGel fusion constructs in tissue culture medium and their subsequent intracellular accumulation after endocytosis was assessed by Western blot analysis using an anti-rGel antibody (Figure 4A). The rGel fusion peptides were added to 2008 cells growing in RPMI medium containing 5% fetal bovine serum for a period of 6 h at a concentration of 1 μM, all the fusion proteins stayed largely intact in the medium without significant degradation suggesting that little or no proteolytic cleavage occurred outside the cultured cells. However, only CPE-R_9_-rGel and R_9_-rGel were detected inside the cells after a 6 h exposure indicating that R_9 _promotes cellular uptake of the cargo molecule. This result indicates that, in the absence of the charge neutralization provided by the E_9_, the R_9 _moiety markedly enhances the cellular accumulation of rGel, but that addition of the E9 sequence is quite effective at blocking this effect. Of note is the fact that the vast majority of the intracellular rGel remained undegraded.

Cellular accumulation of the various fusion proteins was also analyzed by immunofluorescent staining (Figure 4B). The ovarian 2008 cells were exposed to each test protein at a concentration of 250 nM for 6 h at 37°C and, following fixation, they were stained with an anti-gelonin antibody followed by a secondary Texas red-conjugated antibody prior to examination on a deconvoluting microscope. Figure 4B shows the relative amount and intracellular distribution of rGel following incubation with rGel, CPE-G_4_S-rGel, and R_9_-rGel. Control cells not exposed to any of the proteins showed no intracellular staining (data not shown), as was also the case for cells exposed to rGel. Cells exposed to CPE-G_4_S-rGel contained a small amount of dispersed punctuate intracellular staining suggestive of localization within vesicular structures. In contrast, extensive diffuse intracellular staining indicative of cytosolic localization was observed in the cells exposed to R_9_-rGel. Thus, as detected by the immunofluorescent staining, CPE was able to enhance the cellular accumulation of rGel despite the fact that this could not be detected by Western blot analysis. However, consistent with the Western blot analysis, CPE was far less effective than the R_9 _moiety.

### *In vitro *cytotoxicity

**Figure 5:**
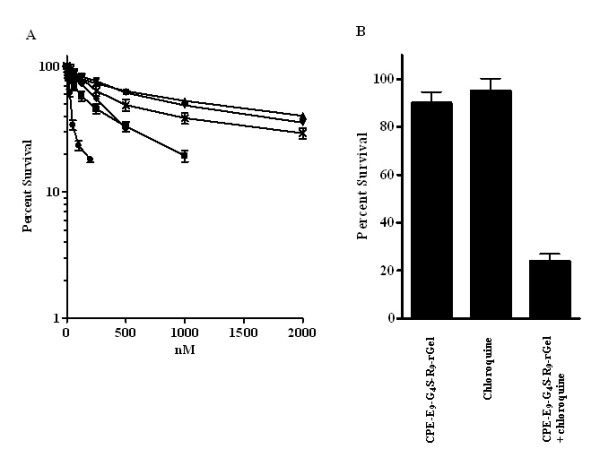
**Cytotoxicity of rGel-based fusion toxins**. **A**. *In vitro *cytotoxicity of various rGel-based series fusion toxins against 2008 cells (solid upright triangle, rGel; solid up-side-down triangle, CPE-G_4_S-rGel; solid circle, R_9_-rGel; solid diamond, CPE-R_9_-rGel; solid sqaure, E_9_-G_4_S-R_9_-rGel; and ×, CPE-E_9_-G_4_S-R_9_-rGel). **B**. Enhancement of the cytotoxicity of CPE-E_9_-G_4_S-R_9_-rGel to 2008 cells by the endosomolytic reagent chloroquine. Cells were treated with 31.25 nM CPE-E_9_-G_4_S-R_9_-rGel for 6 h, washed with complete medium 3 times and subsequently incubated with 50 μM chloroquine for 16 h. Afterwards, the chloroquine-containing medium was also removed and replaced with fresh growth medium after 3 times washing. The cytotoxic effects were determined by CCK-8 assay at 72 h after exposure to CPE-E_9_-G_4_S-R_9_-rGel.

The cytotoxicity of the various fusion proteins was assessed by testing their ability to slow the growth of the 2008 cells. Cells were cultured for 72 h with increasing concentrations of each purified fusion protein and the percent survival in comparison to controls treated only with phosphate buffered saline was determined in CCK-8 assays. As shown in Figure 5A, despite the ability of the CPE peptide to enhance rGel uptake as detected by the immunofluorescent staining, CPE-G_4_S-rGel produced no greater reduction in survival than rGel itself. This indicates that the additional amount of rGel brought into the cell through the interaction of CPE with claudins 3 and 4 was sequestered in an intracellular compartment from which it could not escape to reach the ribosome and inhibit protein synthesis.

Strikingly, when the R_9 _moiety was added to the fusion protein it enhanced cell killing 10-fold over that attained with CPE-G_4_S-rGel alone (IC_50 _78 nM versus 715 nM). To determine whether the enhanced cytotoxic activity of CPE-R_9_-rGel was still dependent on the specific binding of CPE to the claudin receptors on the cell surface, R_9_-rGel was included in the cytotoxicity study. Intriguingly, R_9_-rGel without the CPE targeting moiety displayed even more potent cell killing with an IC_50 _of 38 nM. This is consistent with the well known ability of arginine-rich peptides to efficiently translocate cargo through cell membranes. However, the addition of the R_9 _moiety clearly did not assist in the goal of achieving claudin-dependent cytotoxicity.

### Neutralization of R9 to enhance specificity

Given that the R_9 _moiety was so effective at enhancing uptake, we reasoned that it would likely also be good at releasing rGel from the compartment into which CPE delivered it. However, to make use of the R_9 _sequence in this manner it is necessary to mask the R_9 _until after CPE-mediated and claudin-dependent endocytotic uptake. The addition of a 9 poly-glutamic acid sequence (E_9_) to the R_9 _moiety so as to neutralize its positive charge has previously been shown to disable the ability of R_9 _to enter cells [18]. We explored the use of this approach to block R_9 _non-specific cell membrane penetration until the toxin had undergone internalization mediated by the ligand-receptor binding and ended up in endosomes. The first step was to prove that E_9 _could in fact mask the cell-penetrating capacity of the R9 in 2008 cells. To this end an E_9_-G_4_S-R_9_-rGel fusion protein was produced and tested for cytotoxicity. The addition of the E_9 _through a G_4_S linker to R_9_-rGel increased the IC_50 _by 5.3-fold from 38 nM to 202 nM indicating that the inhibitory E_9 _domain can largely neutralize the R_9 _domain (Figure 5A). Addition of CPE to the E_9_-G_4_S-R_9_-rGel fusion protein further reduced the IC_50 _to 482 nM consistent with the concept that the CPE directs the CPE-E_9_-G_4_S-R_9_-rGel to intracellular vesicles from which it has difficulty escaping. To confirm this, the 2008 cells were incubated with an IC_10 _concentration of CPE-E_9_-G_4_S-R_9_-rGel (31.25 nM) for 6 h and then exposed to a non-toxic concentration (50 uM) of the endosomolytic reagent chloroquine for 16 h. As shown in Figure 5B, addition of chloroquine increased the inhibition of growth produced by this concentration of CPE-E_9_-G_4_S-R_9_-rGel from 10% to 76%. Thus, CPE provides targeting to claudin-expressing tumor cells, R_9 _provides a mechanism for getting out of intracellular compartment, and E_9 _offers an approach to masking the non-specific toxicity of R_9_-rGel. However, a mechanism for cleaving the CPE-E_9 _fragment from the R_9_-rGel fragment once inside the cell is still needed.

### Protease-dependent cleavage

One approach to separating CPE-E_9 _from R_9_-rGel after endocytosis is to introduce a sequence between the E_9 _and R_9 _that is cleavable by a protease found in the compartment into which CPE sequesters the fusion protein. Despite the fact that the R_9 _sequence is itself a substrate for the endosomal/lysosomal protease furin, clearly this reaction was insufficient to release significant amounts of R_9_-rGel. To this end, a series of fusion proteins were produced containing sequences previously reported to be substrates for various endosomal/lysosomal proteases. In an attempt to make the R9 sequence itself a better protease substrate the R9 was modified to the optimum furin-cleavable sequence RRKRRRRRR. In a second attempt, the G_4_S linker was replaced with a GFLG sequence that is cleavable by endosomal cathepsin B. Finally, both were introduced into a third version of the fusion protein as CPE-E_9_-GFLG-R_2_KR_6_-rGel. However, when tested against 2008 cells, none of these further modifications significantly enhanced the cytotoxicity of the CPE-E_9_-G_4_S-R_9_-rGel. We then tried inserting a 10-residue furin cleavable sequence from the diphtheria toxin translocation domain (A_187_GNRVRRSVG_196_, Fdt) between the CPE fragment and the rGel. A recombinant immunoproapoptotic protein containing this sequence was previously shown to result in more potent cell killing activity than those with other furin-sensitive sequences [17]. However, introduction of the Fdt sequence previously reported to enhance endosomal escape also failed to enhance the cytotoxicity of CPE-rGel.

## Discussion

Claudin 3 and 4 are of particular interest as targets for the delivery of protein toxins because they are both consistently over-expressed on some types of tumors and, once bound to their ligand, are rapidly internalized by an endocytotic process. A fusion toxin in which the protein synthesis inhibitory factor (PSIF) was attached to a C-terminal fragment of CPE (C-CPE) was capable of inducing cytolysis in CLDN3/4-expressing MCF-7 human breast cancer cells [10] implying that C-CPE-PSIF must have entered the cytosol. However, the work reported here has exposed some limitations to this approach primarily related to the challenge of getting toxic proteins out of endosomal compartments. We opted to explore CPE-based targeting using the protein toxin gelonin since the requirement for gelonin to escape endosomal/lysosomal compartments to reach the ribosome is well-established, and this toxin has been successfully used to make a variety of immunotoxins in the past [2,14,23,24].

The various fusion proteins tested turned out to be quite stable in the presence of cells and tissue culture media, and those that did get into the 2008 cells were not extensively degraded intracellularly. We have previously documented that the 2008 human ovarian cancer cells express claudin 3 and 4 at levels that are readily detectable by Western blot analysis [12]. Despite the fact that the addition of CPE did not enhance the cellular accumulation of rGel as measured by Western blot analysis, enhanced uptake relative to native rGel was clearly detected by immunofluorescent staining with an anti-gelonin antibody. The pattern of dispersed punctuate staining is consistent with accumulation in the endosomal/lysosomal compartment. However, this degree of enhancement failed to increase the cytotoxicity of the rGel, suggesting that the rGel could not escape from the compartment into which CPE delivered it. The fact that the treatment with a low concentration of chloroquine, which permeabilizes endosomes, enhanced the toxicity of CPE-E_9_-G_4_S-R_9_-rGel provides a second line of evidence that CPE delivers rGel into a subcellular compartment from which it cannot readily escape. It is noteworthy that chloroquine, the anti-malaria drug clinically used in humans, can also be applied to achieve endosome disruption *in vivo *without severe side effects [25], thus holding promise for further optimization and development as a combined therapy with CPE-based toxins.

Arginine-rich peptides have been extensively used to enhance cellular uptake of various types of cargo, and there is a reasonable presumption that they can deliver cargo out of subcellular compartments as well although there is little information on this point. When R_9 _was added to either rGel alone or the CPE-G_4_S-rGel fusion protein it markedly increased uptake and cytotoxicity. However, it also obliterated the selectivity afforded by the CPE component (data not shown). In an attempt to mask the R_9 _sequence until the CPE had successfully delivered the rGel into the cell, we utilized the strategy of neutralizing the charge on R_9 _with an E_9 _sequence previously demonstrated by Tsien and colleagues [18,26]. In this approach the tumor selectivity is achieved by including a sequence between the R_9 _and E_9 _that is cleaved by a matrix metalloproteinase to remove the negatively charged E_9 _glutamate sequence allowing subsequent tumor penetration of a cargo by R_9_. Our design differs in that the recruitment of the fusion toxin to tumor cells is mediated through ligand-receptor interaction, R_9 _provides a mechanism for getting out of intracellular compartment and E9 offers an approach to masking the non-specific toxicity of R_9_-rGel. The addition of E_9_, attached via a flexible G_4_S linker, did indeed substantially reduce the toxicity of R_9_-rGel and CPE-R_9_-rGel confirming in this model the observation that the electrostatic interaction between the E_9 _and R_9 _sequences when they are tethered together disables the ability of R_9 _to translocate cargo across lipid membranes [18,26]. However, despite the fact that the R_9 _sequence itself is a substrate for the endosomal/lysosomal protease furin, it appears that R_9_-rGel was not released from CPE-E_9_-G_4_S-R_9_-rGel after cellular uptake. Given the great potency of R_9_-rGel, one would expect even a modest release to be detected as an increase in cytotoxicity. Interestingly, neither a modification of the R_9 _sequence directed at enhancing its cleavage by furin nor the use of a GFLG linker increased the cytotoxicity of rGel delivered into the intracellular compartment to which it is directed by CPE. Thus, if the use of R_9 _to enhance release of rGel or other protein toxins from intracellular compartment is to be successful, a sequence that is more readily cleaved needs to be found or another approach to linking the E_9 _to the R_9 _developed. Importantly, the CPE-E_9_-linker-R_9_-rGel proteins provide an excellent model system with which to test other types of linkers.

## Conclusions

The results of the studies reported here are relevant to the broad challenge of finding ways of getting therapeutic molecules out of intracellular compartments. There are now a myriad of ways of enhancing the endocytosis of various toxins including attachment to antibodies and ligands for receptors of many types. However, endosomal/lysosomal entrapment and release remains poorly defined. Control of this step offers a substantial opportunity for the development of novel cancer therapeutics that traverse the endosomal/lysosomal compartment of tumor cells. Antibodies to the extracellular domain of CLDN3 and CLDN4 have now been developed [27,28] to which rGel could potentially be linked and it is possible that endocytosis of an antibody rGel complex would deliver rGel into a compartment from which it is more readily released.

## Competing interests

The authors declare that they have no competing interests.

## Authors' contributions

XL and SBH participated in the design of the study and wrote the manuscript. XY, XL and GM carried out the experiments. All authors read and approved the final manuscript.

## Pre-publication history

The pre-publication history for this paper can be accessed here:

http://www.biomedcentral.com/1471-2407/11/61/prepub
